# Detecting Redox Potentials Using Porous Boron Nitride/ATP-DNA Aptamer/Methylene Blue Biosensor to Monitor Microbial Activities

**DOI:** 10.3390/mi13010083

**Published:** 2022-01-04

**Authors:** Kai Guo, Zirui Song, Gaoxing Wang, Chengchun Tang

**Affiliations:** School of Materials Science and Engineering and Hebei Key Laboratory of Boron Nitride Micro and Nano Materials, Hebei University of Technology, Tianjin 300130, China; szr19961019@163.com (Z.S.); gaox202112@163.com (G.W.)

**Keywords:** redox potential, aptamer, dysbiosis, boron nitride, fluorescence

## Abstract

Microbial activity has gained attention because of its impact on the environment and the quality of people’s lives. Most of today’s methods, which include genome sequencing and electrochemistry, are costly and difficult to manage. Our group proposed a method using the redox potential change to detect microbial activity, which is rooted in the concept that metabolic activity can change the redox potential of a microbial community. The redox potential change was captured by a biosensor consisting of porous boron nitride, ATP-DNA aptamer, and methylene blue as the fluorophore. This assembly can switch on or off when there is a redox potential change, and this change leads to a fluorescence change that can be examined using a multipurpose microplate reader. The results show that this biosensor can detect microbial community changes when its composition is changed or toxic metals are ingested.

## 1. Introduction

Microbial activities reveal environmental conditions; for example, Caribbean reefs are over-run with infectious diseases caused by microbes, which stresses the urgent need to evaluate microbial activities in a timely manner [1]. To detect microbial activity with high accuracy, microbial adenosine is measured using a somewhat complex and expensive kit [2]. However, there are other indicators of microbiomes. The fascinating fact is that bacteria themselves are sensitive to oxidative stress, which can impact the metabolic system [3]. Therefore, it is imperative to gain adequate knowledge of how environmental factors can interact with the bacterial redox sensor system. In other words, rapid detection of redox changes in microbiomes in environmental niches provides a means to rapidly detect dysbiosis.

Aptamers are nucleic acid fragments that grow in vitro and are sensitive to various molecules, including biological molecules and environmental toxins. Using the Systematic Evolution of Ligands by Exponential Enrichment (SELEX) protocol, aptamers can be selected for molecules of interest [4], such as thrombin, a critical protease of blood coagulation [5]. Many taxa of bacteria identify membrane proteins as receptors that have a strong affinity to aptamers. Thus, aptamers are great tools for identifying bacteria, such as *Staphylococcus aureus* [6,7]. In addition, aptamers are a great tool for detection because of their sharp color differentiation [6].

RNA aptamers have been developed as “light-up” probes whose sharp fluorescence signal can facilitate many applications, and the susceptibility of RNA restricts its implementation in vitro [8]. However, DNA aptamers are more stable and photochemically detectable when coupled with instruments such as a flow cytometer [9]. Additionally, its unique structure—one strand as a probe and another as an optical site—allows it to illuminate targets [10]. An adenosine aptamer associated with gold nanoparticles has been found to illuminate samples containing adenosine and cocaine [11]. In particular, these intricate designs have inspired new routes to simplify the assembly of aptamers and fluorophores; for example, a single-strand DNA aptamer was applied to detect different redox environments [12]. MB was connected to the intra sites of DNA grooves when it was in an oxidized environment; it was off the DNA loop when it was in a reduced environment. However, this study did not consider the changes in the fluorescence of MB. Hypothetically, fluorescence changes when there is a conformational change in the fluorophore. The fluorescence change, either intensity change or emission spectra change, can manifest the environmental condition; for example, high redox indicates adequate oxidative reactive species (ORS) level, which is accompanied by respiratory virus infection [13]. To enhance and stabilize the signal, 2D nanomaterials, such as graphene [14], carbon nanotubes [15], and gold [16,17], have been applied as the core for biosensors. Hexagonal boron nitride—the “white graphene”—is structurally identical to graphene, and it has already been found to have tunable electrical properties and excellent fabrication capacities [18]. BN nanosheets have been applied to synthesize the “sandwich” immunosensor with gold and graphene [19]. pBN is another form of BN nanofiber, which can be regarded as a good candidate for a fluorophore quencher. Upon reviewing the literature to date, we found no report of the use of pBN in the fabrication of biosensors.

In the present study, we established a correlation between the fluorescence and redox potential. The sensitivity and selectivity of this approach makes it possible to detect dysbiosis.

## 2. Materials and Methods

The ingredients used to synthesize pBN, namely melamine (C_3_N_6_H_6_) and boric acid (H_3_BO_3_), were obtained from Aladdin Biochemical Technology Co., Ltd.

**Figure 1:**
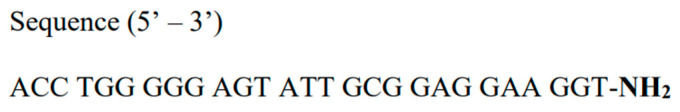
DNA base sequence of the chosen aptamer.

A series of reagents were used to synthesize the biosensor assembly. The DNA aptamer (Figure 1) was obtained from Shanghai Sangon Biotech Co. (Shanghai, China) Adenosine 5-triphosphate disodium salt (ATP) was obtained from Macklin Biochemical Co., Ltd. (Shanghai, China). Poly (diallyldimethylammonium chloride) (PDDA) (20 wt.% in H_2_O) and MES to prepare buffer solution (10 mM) were purchased from Rhawn Co. Ltd. HEPES (Shanghai, China) was obtained from Solarbio Ltd. (Beijing, China) to prepare a 10 mM buffer solution. 1-(3-dimethylaminopropyl)-3-ethylcarbodiimide (EDC) was purchased from J&K Chemicals Co. Ltd. (Shanghai, China). In addition, 1-hydroxypyrrolidine-2,5-dione (NHS) was purchased from Bidepharm Co. Ltd. MB (Shanghai, China) was obtained from Fuchen Chemical Co, Ltd. (Tianjin, China).

Two reagents, FeCl_3_·6H_2_O (Damao Chemical Factory, Tianjin, China) and ascorbic acid (J&K Scientific, Beijing, China), were used for the redox potential sensitivity study.

For the microbial study, the bacterial strain *Escherichia coli* (*E. coli*) and *Bacillus subtilis* were obtained from Beijing Sanyao Science & Technology Development Co (Beijing, China). In addition, *Flavobacterium* sp. in a frozen tube was obtained from the China Centre for Type Culture Collection (Wuhan, China). To prepare the culture media, R2A liquid medium and nutrient broth were purchased from Qingdao Hope Biotechnology Co., Ltd. (Qingdao, China).

To prepare the biofilm, anhydrous calcium chloride (CaCl_2_) was obtained from Kermel Chemical Co., and sodium alginate ((C_6_H_7_NaO_6_)_n_) was obtained from Tianjin Fengchuan Chemical Reagent Technologies Co. Ltd. (Tianjin, China).

The heavy metals used to induce toxicity in the microbial community were Zn (CH_3_COO)_2_ (Fengchuan Chemical Reagent Technology Co. Ltd., Tianjin, China), PbCl_2_ (Damas-beta Chemical Co. Ltd., Shanghai, China), Cs_2_CO_3_ (Macklin Biochemical Co. Ltd., Shanghai, China), and CoCl_2_·6H_2_O (Rhawn Co. Ltd., Shanghai, China).

To test the redox potential, a portable ORP device (ORP-2, Beijing Shunkeda Technology Co. Ltd., Beijing, China) was used.

The multipurpose microplate reader, Molecular Devices i3x, was used to examine fluorescence when the biosensor was added to the opaque 96-well microplate.

IR spectra were collected using a Bruker TENSOR 27 FTIR spectrometer. The zeta potential was examined using a PSS-Nicomp 380 particle sizer. A laser scanning confocal microscope (OLYMPUS OLS5000-LAF) was used to obtain images of the biosensor. Flow cytometry was performed using a FACSAria SORP (Becton Dickinson, Franklin Lakes, NJ, USA). 16S rRNA sequencing was performed by Berry Genomics, China, Ltd. (Beijing, China).

The synthetic route for pBN was based on Song’s 2020 study [20]. Melamine, measured to 21 g, along with 20.6 g boric acid, were added to 1 L deionized water. The mixture was then stirred and heated at 80 °C for 4 h, after which it was allowed to naturally cool and crystallized. The product was then vacuum dried, and the precursor was obtained. Next, in a tubing furnace, the precursor was placed and heated in N_2_ atmosphere to 1050 °C for 240 min, and then kept for 4 h. After natural cooling to room temperature, the product became pBN.

PDDA (0.6 mL, 20 wt% in H_2_O), and pBN (40 mg) were added to 100 mL of ultrapure H_2_O, and the mixture was sonicated for 5 h [19]. The suspension was then centrifuged at 50,000× *g* for 30 min to obtain the white precipitate (the hydrolyzed pBN), which was dried at 80 °C for 4 h [21]. Next, 30 mg of the as-prepared pBN was added to 200 mL of HEPES under sonication for 30 min. A 5-mL volume of the suspension was then measured and labeled as A. Next, the ATP-aptamer/dye assembly was constructed. In a 10-mL centrifuge tube (labeled as B) with 5 mL MES buffer (10 mM), 100 µL aptamer (Figure 1), 100 µM ATP, 200 µM MB, 500 µM EDC, and 500 µM NHS were added. Finally, A and B were mixed and swirled in a rotator for 40 min, thus producing the cross-linking form of the aptamer.

To test how the aptamer responded to different redox environments, a simplified biosensor without integrating pBN was used. This biosensor solution was added to 1 mL of ascorbic acid or Fe (III) solution (0.00001 M, 0.0001 M, 0.001 M, and 0.01 M), which were then mixed well and tested using Fluorolog Horiba (FL3-22) to obtain the fluorescence intensity.

*Flavobacteria* sp. were grown in R2A liquid culture for 18 h at 18 °C and *E. coli* and *Bacillus subtilis* were grown in nutrient broth for 18 h at 37 °C. When the OD_630_ reached approximately 0.4 for each species, the following procedures were performed. A volume of 3 mL of *Flavobacteria* sp. fluid was added to each of 22 testing tubes. Subsequently, volumes of 0, 100, 200, 300, and up to 1000 µL *E. coli* or *Bacillus subtilis* fluids were added to each test tube with two replicates for each sample. Afterwards, all samples were placed on a shaker for another 18 h at 18 °C. The OD_630_, pH, and ORP were then measured for each sample.

Biofilm making is a way to simulate how microbial species inhabit tissues [22]. In the current study, a biofilm-fluorescence assay was freshly prepared and tested using a multipurpose microplate reader (SpectraMax i3x). In a dark 96-well plate, 20 µL CaCl_2_ (50 mM), 20 µL alginic acid (4 g·100 mL^−1^ H_2_O), microbes with different volumes (Figure S1) from the last step, and 1 µL biosensor from step 2 were added to each well. The plate was then placed into a microplate reader (25 °C, orbital shaking for 30 s before testing, excitation wavelength of 565 nm, and emission wavelength of 665 nm) to test the fluorescence intensity.

In a separate experiment, the toxicity induced by heavy metals in each microbial community was examined using a microplate reader. In a dark 96-well plate, 20 µL of CaCl_2_ (50 mM), 20 µL of alginic acid (4 g·100 mL^−1^ H_2_O), microbes including *E. coli*, *Flavobacterium* sp., and *Bacillus subtilis* with the same volume (50 μL each) from the last step, heavy metals, namely Zn (CH_3_COO)_2_ (i), Cs_2_CO_3_ (j), CoCl_2_ (k), and PbCl_2_ (l) with different volumes (Figure S2), and 1 µL biosensor from step 2 were added to each well. The plate was then placed into a microplate reader (25 °C, orbital shaking for 30 s before testing, excitation wavelength of 565 nm, and emission wavelength of 665 nm) to test the fluorescence intensity.

## 3. Results

### 3.1. Characterization of the Biosensor

Upon applying the FTIR technique, we found characteristic peaks from samples including pBN, MB, and the biosensor we synthesized. The confirmation of the interaction between pBN, MB, and the DNA aptamer was evidenced by the different spectra shown in Figure S3: isolated pBN, MB, and the assembled biosensor.

After measuring the zeta potential, the biosensor was tested at 13 mV, which is high enough to indicate the dispersibility of the composite. Another sample tested was the one containing *E. coli*, *Flavobacterium* sp., and *Bacillus subtilis*, in which the biosensor was added, and for which the zeta potential was tested as −32 mV.

The biosensor has a layered structure (Figure S4a). Figure S4b depicts the interaction between *Flavobacterium* sp. and the biosensor, with a fascinating impression of waves on the ocean. The more complex the system is, the more complex the color and appearance we see in the image; in Figure S4c, the interaction between *Flavobacterium* sp., *E. coli*, *Bacillus subtilis*, and the biosensor is illustrated.

### 3.2. Biosensor in Environment with Various Redox Potentials

Interesting colorimetric results were obtained after 1 mL MB/biosensor was added to 4 mL of a solution with different redox potentials. This color differentiation can be seen in Figure S5: (a) MB; (b) MB/biosensor; (c) MB/biosensor-0.01M Fe (III); (d) MB/biosensor-0.01M ascorbic acid.

The MB/aptamer biosensor acts as a sensitive tool for a strongly oxidized environment (Figure S6).

When the concentration of Fe (III) was increased, the redox potential increased, and the fluorescence intensity decreased. When all three variables were plotted on the same map, a unique symmetrical “mirror” image was obtained (Figure S7). This is an important finding, because dysbiosis can induce oxidative stress, as measured by redox potential. Now, by observing the fluorescence change, we obtain first-hand knowledge of the health condition of the species.

### 3.3. Biosensor Performance in Various Microbial Fluids

Growing *Flavobacterium* sp. and *E. coli/Bacillus subtilis* together, OD_630_ was measured (Figure S8).

The bacteria that grew in the liquid culture were aerobic. A small amount of lactic acid was excreted into the media owing to the anaerobic process. Thus, we observed an overall decline in pH (Figure S9).

**Figure 2:**
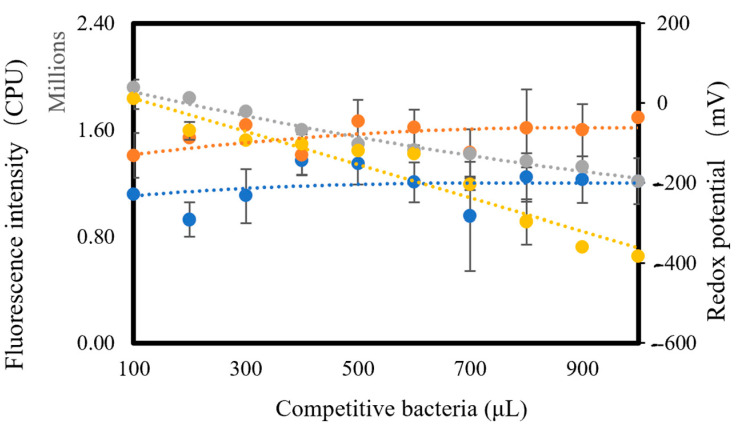
The inversely proportional relationship between fluorescence intensity and redox potential (blue line: fluorescence after adding *E. coli*; orange line: fluorescence after adding *Bacillus subtilis*; grey line: redox potential after adding *Bacillus subtilis*; yellow line: redox potential after adding *E. coli*).

Adding more competitive bacterial strains, *E. coli* or Bacillus *subtilis*, to the major bacterium, *Flavobacterium* sp., resulted in the decline of oxidative reactive species (ORS) in the media, which led to a decline in redox potential (Figure 2). However, we observed an overall increase in the fluorescence intensity.

**Figure 3:**
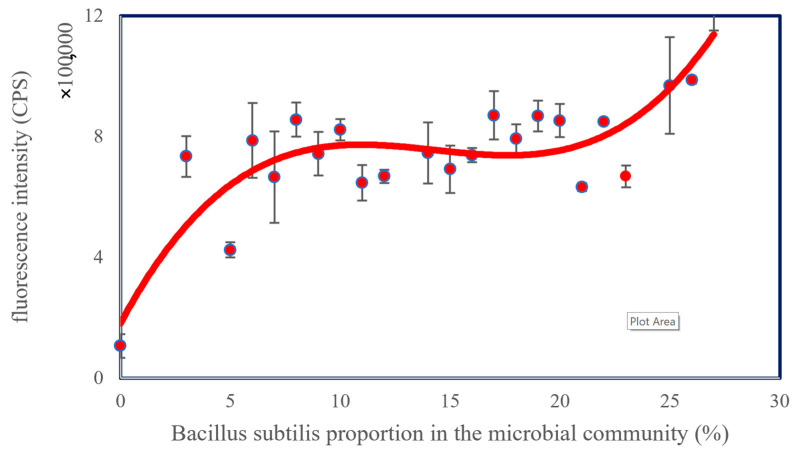
The trend of fluorescence intensity at the initial stage of microbial composition.

After preparing the community of *Flavobacterium* sp., *E. coli*, and *Bacillus subtilis*, changing the proportion of each species can alter the fluorescence intensity. In this study, *Flavobacterium* sp. was the major species. When the proportion of *Bacillus subtilis* increased to above 20%, an increase in fluorescence intensity was observed, as shown in Figure 3.

**Figure 4:**
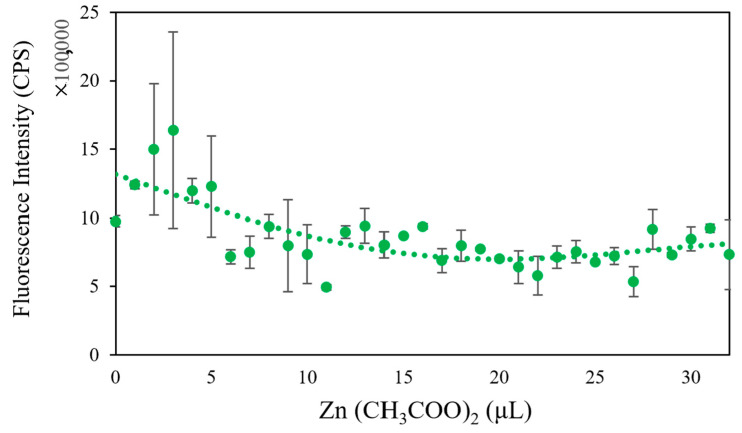
The trend of fluorescence intensity when changing the amount of added Zn (CH_3_COO)_2_.

**Figure 5:**
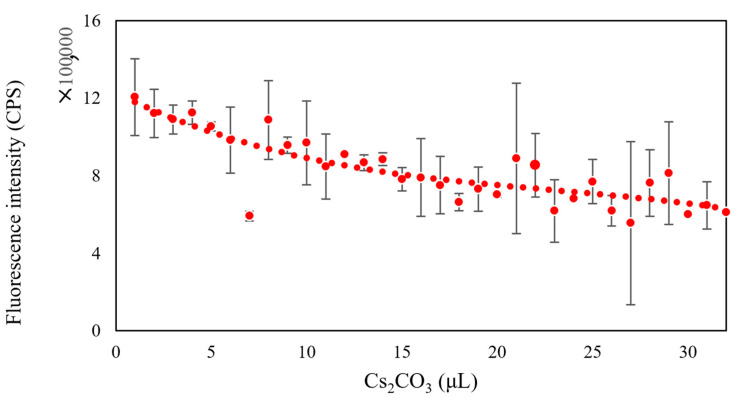
The trend of fluorescence intensity when changing the amount of added Cs_2_CO_3_.

**Figure 6:**
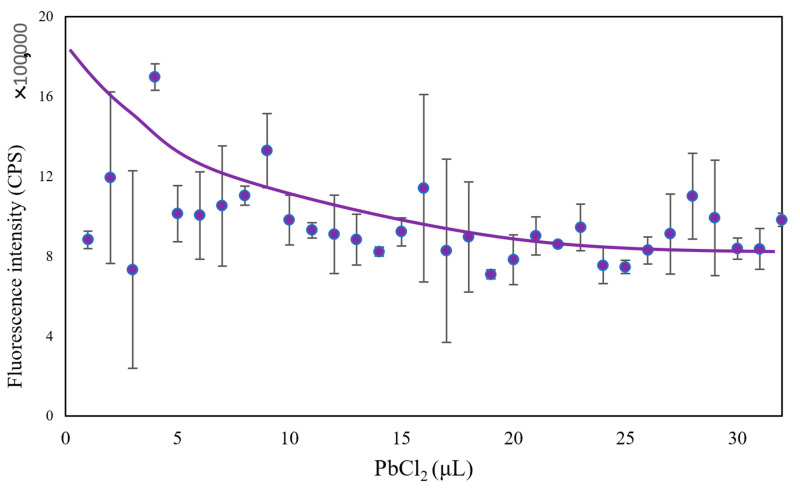
The trend of fluorescence intensity when changing the amount of added PbCl_2_.

**Figure 7:**
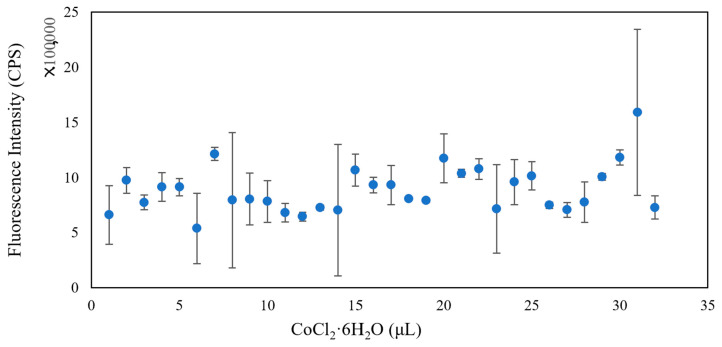
The trend of fluorescence intensity when changing the amount of added CoCl_2_·6H_2_O.

In addition to changing the composition of microbial fluids, we also added heavy metal solutions with different concentrations to the microbial community composed of *Flavobacterium* sp., *Escherichia coli*, and *Bacillus subtilis*. The heavy metals used were Zn (CH_3_COO)_2_, Cs_2_CO_3_, CoCl_2_·6H_2_O, and PbCl_2_. As can be seen in Figure 4, Figure 5 and Figure 6, the fluorescence intensity clearly declines as the volume of added heavy metals increases. However, there was no overall integrated trend for CoCl_2_·6H_2_O (Figure 7).

### 3.4. Using Flowcytometry to Detect the Change of Reactive Oxygen Species (ROS)

**Figure 8:**
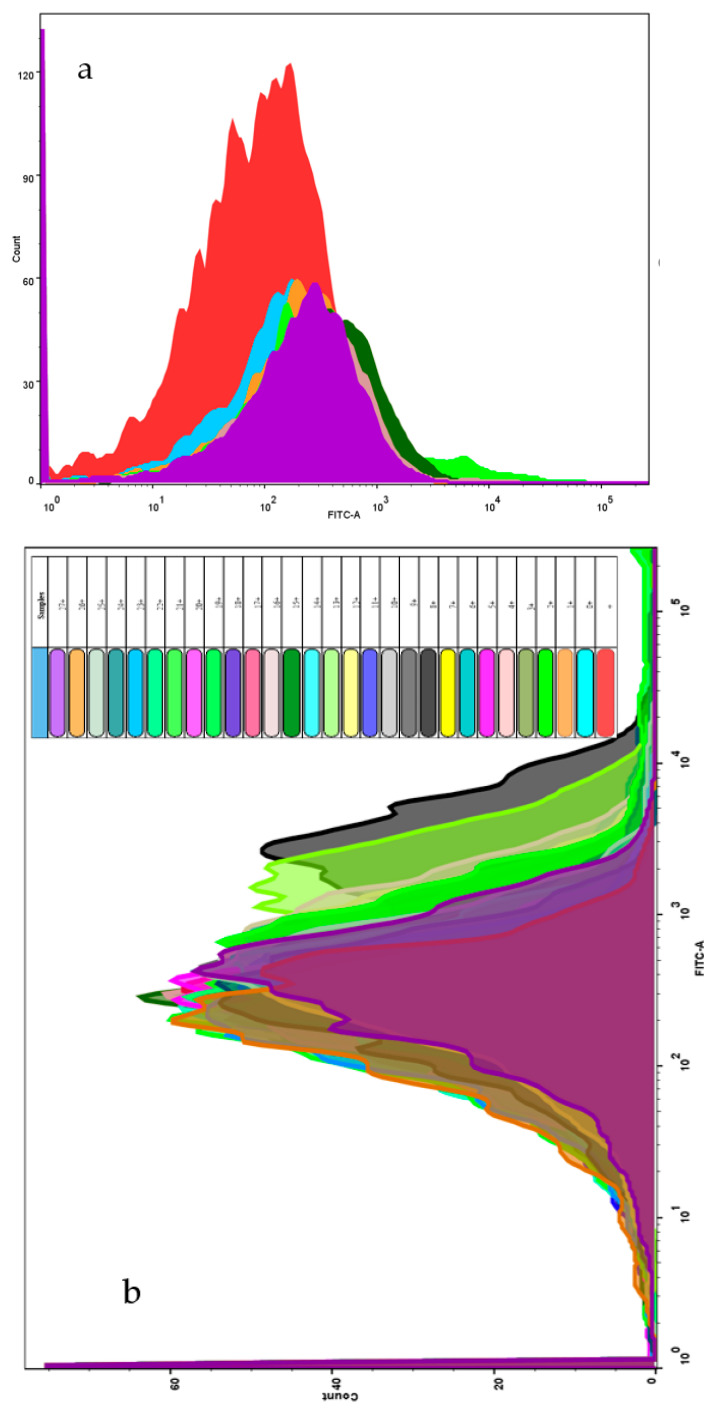
Relative ROS content comparison among two experimental sets: (**a**) bacterial composition change (from 0 to 6.0% to 6% *Bacillus subtilis* was taking the space of each microbial community); (**b**) induced toxicity by heavy metals (“+” is the Rossup positive control, and 0+ to 27+ stand for 0 µL to 27 µL Zn (CH_3_COO)_2_ ingested by each microbial community).

In the last step, we found that the fluorescence changes as the microbial activity changes. To explain this phenomenon, flow cytometry was used to test the change in ROS in microbial fluids (Figure 8).

### 3.5. S rRNA Analysis

**Figure 9:**
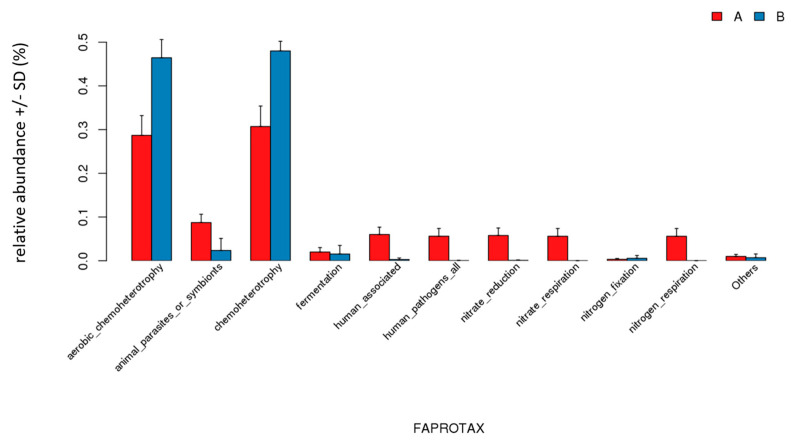
Texo-abundance analysis shows that the heavy metals induced microbial communities have lost part of the metabolic functions.

Overall, the 16S rRNA analysis corresponds to our results obtained from the biosensor (Figure 9, where Group A includes 10 samples GK0-GK9, and Group B includes 10 samples GK10-GK19).

**Figure 10:**
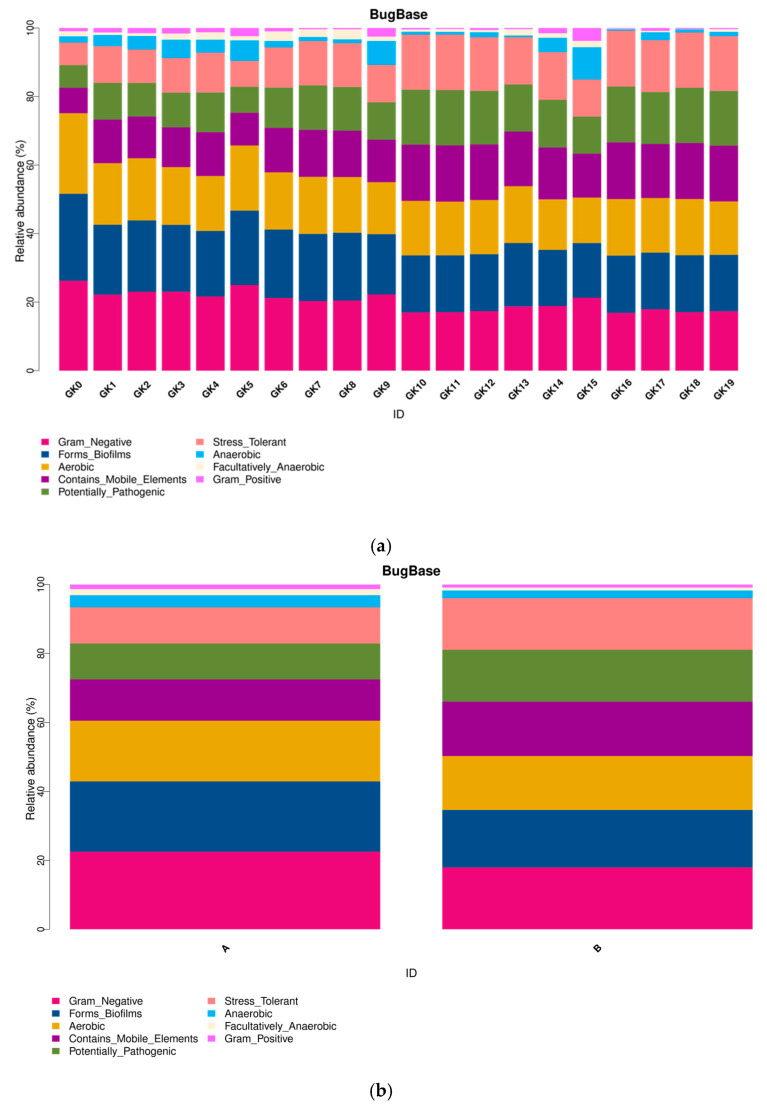
Functional relative abundance of a microbial community. (**a**) distinction of abundance between different treated colonies; (**b**) comparison between composition change of a colony (Group A) and Zn (CH_3_COOH)_2_ added to a colony (Group B).

The bar graph delineates the evolution of each colony for multiple categories (Figure 10).

**Figure 11:**
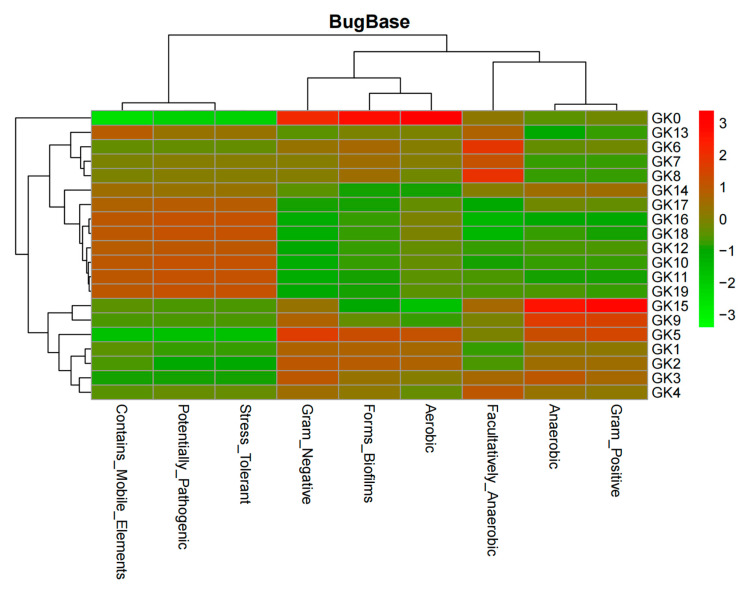
Heatmap depicting physiological activity of a microbial community.

The heatmap (Figure 11) can be read as follows: when the color gets “hotter”, the corresponding action becomes more aggravated; when it gets “colder”, the action worsens.

**Figure 12:**
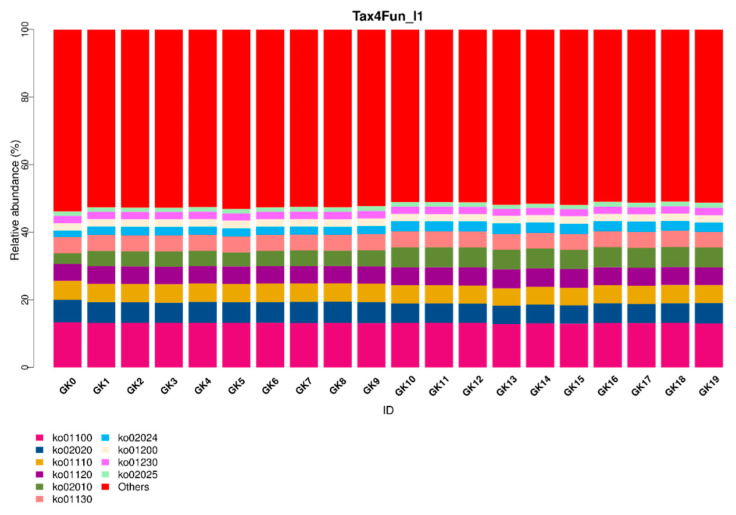
Tax4Fun2 functional prediction of a microbial community.

Using 16S rRNA, multiple pathways such as ATP-binding cassette (ABC) transporters (ko02010), for which the relative abundance is growing, are displayed for all samples GK0-GK19 (Figure 12).

## 4. Discussion

In Figure S3, amine (N–H bending) and aromatic amine (C–N stretching) peaks are shown, which are characteristic peaks of MB. Compared with the peaks in MB, the peaks of pBN are simpler, because of the simple bonding pattern in pBN. The spectra of the biosensor contain peaks belonging to the DNA aptamer. Imine (C=N stretching) groups were observed at ~1637 cm^−1^, which represent base pairs in DNA. Sulfonyl groups (S=O) from the interaction of sulfur in MB and phosphate groups in DNA can be found at ~1385 cm^−1^. Overall, the peaks from the biosensor are a combination of peaks in pBN and MB.

In comparing Figure S5a,b, a lighter blue color in (b) is apparent, due to the addition of the aptamer. This is evidence that MB can interact with DNA grooves to change the color appearance of MB. In Figure S5c,d, light blue is apparent in (c) and clear white in (d). As a strong oxidizer, Fe (III) interacts with MB and the aptamer, resulting in a switchable conformational change between MB and the aptamer, and ascorbic acid, which is conversely a strong reducer. This also proves that MB-ATP-aptamer can switch “on” when it is in an oxidized environment, and switch “off” when it is in a reduced environment [12].

The higher the concentration of Fe (III), the lower the fluorescence intensity (Figure S6). There is an antagonistic effect when MB interacts with concentrated Fe (III), which can absorb emission photons from MB [23]. When MB interacts with the aptamer, there is a π-π stack between the MB and DNA base pairs. A conformational change in MB, the fluorescence probe, causes the shift in emission spectra, from 690 nm to approximately 696 nm [24,25].

In the study of biosensors in an environment with various redox potentials, the MB/aptamer biosensor can demonstrate the colorimetric difference in different redox potential environments, yet it is not sensitive enough to demonstrate the correlation between fluorescence intensity and redox potential in a strongly reduced environment. However, the living conditions for bacteria vary, and the redox potential impacted by ascorbic acid cannot fully manifest, as proved by the microbial study we did next.

Surprisingly, when *Bacillus subtilis*, a Gram-positive bacterium, grew together with *Flavobacterium* sp., a Gram-negative bacterium, the overall OD_630_ was elevated compared with the values from *E. coli*, a Gram-negative bacterium, and *Flavobacterium* sp. (Figure S8). The competition between two different Gram-positive bacteria was not pronounced. It can be explained that the reactivity of *Flavobacterium* sp. was much higher than that of its competitor, *Bacillus subtilis*, and as a result, *Flavobacterium* sp. grew even faster.

This inversely proportional relationship (Figure 2) between the redox potential and fluorescence intensity corresponds with the results shown in Figure S7. Carbon nanotubes [26], nano C60 [27], and magnetic nanomaterials [28] have been developed as fluorescence quenchers. Technically, pBN can be regarded as a fluorescence quencher. Nucleic acids, such as DNA, were tightly adsorbed onto pBN [29]. Furthermore, aptamers are prone to structural switching when encountering any specific target so that the DNA/DNA duplex is converted to a DNA/target complex [10,30], which enhances the selectivity of our biosensor. RNA aptamers can specifically bind to malachite green, a tag, to produce fluorescence [31]; hence, MB is a legitimate option as a tag to release fluorescence in our study. In an oxidized environment, MB was bound to the DNA base-pair zone, which resulted in its close vicinity to pBN; however, in a reduced environment, MB was removed from the aptamer, which furthered its distance to pBN, and consequently, the quenching ability of pBN was reduced. This explains why the lower the redox potential, the higher the fluorescence intensity of this biosensor.

The significant indicator (Figure 3) indicated that the biosensor could detect changes in the microbial community. When the proportion of *Bacillus subtilis* is within 5–20%, the fluorescence levels off, and the fluorescence rises rapidly when the proportion is higher than 20%. In an actual aqueous environment, *Flavobacterium* sp. is usually the major microbial species in fish mucus. In addition, *E. coli* and *Bacillus subtilis* may also be present. As environmental factors change, the composition of each microbial community changes as well. Evidently, using the method in this study, we can detect the changing trend of the microbial community with a micro amount of bacterial fluid, ranging from 100 to 200 µL. A similar method requires a small amount of sample to examine glucosidase activity using a biochar-enhanced enzyme kit [32], which is not cost-effective for long-term monitoring. In addition, operating a potentiometric meter can measure pH or redox potential (Eh) of a microbial culture; however, a high volume of a sample must be taken into account [33]. Even without using expensive methods such as 16S rRNA, we can still have a general idea about the health conditions of microbial communities.

Heavy metals can induce the production of ROS in microbial communities, which helps to elevate the redox potential of microbial communities. Aptamers can sensitively probe foreign substances, such as bisphenol A, in cells when cooperating with gold nanoparticles, a fluorescence quencher [34]. Thus, we tested whether our biosensor could provide optimal signals when various heavy metals invade a microbial community. From the evidence, we already have in the study, the higher the redox potential, the lower the fluorescence intensity. The results indicated that the designed biosensor could track the induced toxicity level in a microbial community. In addition, the biosensor exhibited good selectivity for various heavy metals. When Zn (CH_3_COO)_2_ was added to the microbial community from low amount to high amount, the fluorescence intensity declined from 2.0 × 10^6^ CPS to 1.0 × 10^6^ CPS ($2.0\times 10^{6}/\left(1.0\times 10^{6}\right)=2)\;$(Figure 4); with the same amount of Cs_2_CO_3_ added, the fluorescence intensity declined with increasing Cs_2_CO_3_, from 1.2 × 10^6^ CPS to 6.0 × 10^5^ CPS ($1.2\times 10^{6}/\left(6.0\times 10^{5}\right)=2)\;$(Figure 5); yet, the fluorescence intensity declined from 2.0 × 10^6^ CPS to 8.0 × 10^5^ CPS for PbCl_2_ ($2.0\times 10^{6}/\left(8.0\times 10^{5}\right)=2.5)\;$(Figure 6). The changing range of fluorescence intensity (Figure 7) was maintained at a similar scale.

According to flow cytometry analysis, as the proportion of *Flavobacterium* sp. declines, the collected ROS shifts more to the right, which indicates that as other bacterial species join the community, *Flavobacterium* sp. must compete with them to produce more ROS (Figure 8a). In another set of studies, Zn (CH_3_COO)_2_ was added to the microbial community. The ROS content was shifted to the right as more Zn (CH_3_COO)_2_ was added, which is a clear indicator that Zn (CH_3_COO)_2_ can induce toxicity to the microbial community (Figure 8b). The results confirm those in Figure 4, where the fluorescence intensity declines as the redox potential increases.

Lastly, the 16S rRNA technique (Figure 9, Figure 10, Figure 11 and Figure 12) further confirmed the accuracy of our biosensor. GK0-GK9 contains *Flavobacteria* sp., the major species, and *Bacillus subtilis*, the competition species, which occupies an increasing amount of space in the microbial colony. In addition, GK10-GK19 stands for the colony filled with the same species, but different amounts of Zn (CH_3_COOH)_2_. Because the ratio of *Flavobacteria* sp. was compressed and that of *Bacillus subtilis* was inflated from GK0-GK9, the bar representing Gram-negative bacteria contracts, which represents the expansion of Gram-positive bacteria. The pinkish-orange bar denoting the stress tolerance is distinctively longer for samples GK10-GK19 owing to their stimulation by Zn (CH_3_COOH)_2_. GK0 represents the purest colony so that it tends to form the most stabilized biofilm, which is represented by the brightest red color. From GK1-GK9, more competitive bacterial species were added, which allowed the biofilm to become more fragile, as can be seen that the colder color deepens. The greenish color of samples GK10-GK19 indicates that it is too difficult for microbial biofilms to form, since the heavy metal intake is increasing owing to the distinctive reddish color grids representing mobile elements. The biosensor we synthesized can intricately predict the metabolic and physiological activities of a microbial colony.

To date, 16S rRNA is still the best tool for predicting and elucidating various metabolic activities. The growing trend for the ATP-binding cassette (ABC) transporters (ko02010) pathway is a result of the intensified stimulators to a microbial community. The drawback of this method is that cross-contamination within a microbial community with low biomass is inevitable [35]. However, the advantage of our biosensor is that the abnormal and alarming activities of a microbial community can be detected rapidly. The information we report here provides a foundation for further study, in which a more comprehensive database will be established. We aim to provide practical results that are of value in both environmental and medical applications, especially where microbial activity is a research focus.

## 5. Conclusions

The overall study confirms that the biosensor can detect microbial changes without the assistance of electrochemical devices. In binary or tertiary microbial systems, there is a clear inversely proportional correspondence between fluorescence and redox potential. Interestingly, the biosensor can detect the microbial activities under the influence of heavy metals, given the confirmed inversely proportional relationship between fluorescence and redox potential. Using this biosensor can track dysbiosis in much broader environmental matrices.

## Supplementary Materials

The following are available online at https://www.mdpi.com/article/10.3390/mi13010083/s1, Figure S1: Fluorescence testing when changing the composition of the microbial community (a: *Flavobacteria* sp.; b: *E. coli*; c: *Bacillus subtilis*). Figure S2: Heavy metals added to each well (Control A1 and A2). Zn (CH_3_COO)_2_ (i) is shown as an example here, according to which the other three metals are added as well. Figure S3: FTIR spectra of the biosensor, pBN, and MB. Figure S4: Images for biosensor (a), *Flavobacterium* sp. and biosensor (b), and *Flavobacterium* sp. and *E. coli.* & *Bacillus subtilis* (c) using a laser bifocal microscope. Figure S5: Colorimetric differences (a) MB; (b) MB-aptamer; (c) MB-aptamer-Fe (III); (d) MB-aptamer-ascorbic acid. Figure S6: Fluorescence change when MB/biosensor was added to Fe (III) solutions with different concentrations. Figure S7: “Mirror” relationship between fluorescence intensity and redox potential as the concentration of Fe (III) increased. Figure S8: OD_630_ values after *Flavobacteria* sp. were grown in *E. coli* or *Bacillus subtilis*. Figure S9: pH changes after *Flavobacteria* sp. were grown in *E. coli* and *Bacillus subtilis*.

## References

[1] Harvell D., Jordan-Dahlgren E., Merkel S., Rosenberg E., Raymundo L., Smith G., Weil E., Willis B. (2007). Global Environmental Facility Coral Reef Targeted Research Program Coral Disease, Environmental Drivers, and the Balance between Coral and Microbial Associates. Oceanography.

[2] Hammes F., Goldschmidt F., Vital M., Wang Y., Egli T. (2010). Measurement and Interpretation of Microbial Adenosine Tri-Phosphate (ATP) in Aquatic Environments. Water Res..

[3] Green J., Paget M.S. (2004). Bacterial Redox Sensors. Nat. Rev. Microbiol..

[4] Ellington A.D., Szostak J.W. (1990). In Vitro Selection of RNA Molecules That Bind Specific Ligands. Nature.

[5] Deng B., Lin Y., Wang C., Li F., Wang Z., Zhang H., Li X.F., Le X.C. (2014). Aptamer Binding Assays for Proteins: The Thrombin Example-A Review. Anal. Chim. Acta.

[6] Yu T., Xu H., Zhao Y., Han Y., Zhang Y., Zhang J., Xu C., Wang W., Guo Q., Ge J. (2020). Aptamer Based High Throughput Colorimetric Biosensor for Detection of Staphylococcus Aureus. Sci. Rep..

[7] Shahdordizadeh M., Taghdisi S.M., Ansari N., Alebooye Langroodi F., Abnous K., Ramezani M. (2017). Aptamer Based Biosensors for Detection of Staphylococcus Aureus. Sens. Actuators B Chem..

[8] Ouellet J. (2016). RNA Fluorescence with Light-Up Aptamers. Front. Chem..

[9] Huang P.J.J., Liu J. (2010). Flow Cytometry-Assisted Detection of Adenosine in Serum with an Immobilized Aptamer Sensor. Anal. Chem..

[10] Nutiu R., Li Y. (2003). Structure-Switching Signaling Aptamers. J. Am. Chem. Soc..

[11] Liu J., Lu Y. (2006). Fast Colorimetric Sensing of Adenosine and Cocaine Based on a General Sensor Design Involving Aptamers and Nanoparticles. Angew. Chem..

[12] Biniuri Y., Luo G.F., Fadeev M., Wulf V., Willner I. (2019). Redox-Switchable Binding Properties of the ATP-Aptamer. J. Am. Chem. Soc..

[13] Khomich O.A., Kochetkov S.N., Bartosch B., Ivanov A.V. (2018). Redox Biology of Respiratory Viral Infections. Viruses.

[14] Yang G., Zhu C., Du D., Zhu J., Lin Y. (2015). Graphene-like Two-Dimensional Layered Nanomaterials: Applications in Biosensors and Nanomedicine. Nanoscale.

[15] Zamzami M.A., Rabbani G., Ahmad A., Basalah A.A., Al-Sabban W.H., Nate Ahn S., Choudhry H. (2021). Carbon Nanotube Field-Effect Transistor (CNT-FET)-Based Biosensor for Rapid Detection of SARS-CoV-2 (COVID-19) Surface Spike Protein S1. Bioelectrochemistry.

[16] Kim H., Park M., Hwang J., Kim J.H., Chung D.R., Lee K.S., Kang M. (2019). Development of Label-Free Colorimetric Assay for MERS-CoV Using Gold Nanoparticles. ACS Sens..

[17] Layqah L.A., Eissa S. (2019). An Electrochemical Immunosensor for the Corona Virus Associated with the Middle East Respiratory Syndrome Using an Array of Gold Nanoparticle-Modified Carbon Electrodes. Microchim. Acta.

[18] Zeng H., Zhi C., Zhang Z., Wei X., Wang X., Guo W., Bando Y., Golberg D. (2010). “White Graphenes”: Boron Nitride Nanoribbons via Boron Nitride Nanotube Unwrapping. Nano Lett..

[19] Yang G.H., Shi J.J., Wang S., Xiong W.W., Jiang L.P., Burda C., Zhu J.J. (2013). Fabrication of a Boron Nitride-Gold Nanocluster Composite and Its Versatile Application for Immunoassays. Chem. Commun..

[20] Song Q., Liang J., Fang Y., Guo Z., Du Z., Zhang L., Liu Z., Huang Y., Lin J., Tang C. (2020). Nickel (II) Modified Porous Boron Nitride: An Effective Adsorbent for Tetracycline Removal from Aqueous Solution. Chem. Eng. J..

[21] Sharma M.K., Narayanan J., Pardasani D., Srivastava D.N., Upadhyay S., Goel A.K. (2016). Ultrasensitive Electrochemical Immunoassay for Surface Array Protein, a Bacillus Anthracis Biomarker Using Au-Pd Nanocrystals Loaded on Boron-Nitride Nanosheets as Catalytic Labels. Biosens. Bioelectron..

[22] Stewart E.J., Ganesan M., Younger J.G., Solomon M.J. (2015). Artificial Biofilms Establish the Role of Matrix Interactions in Staphylococcal Biofilm Assembly and Disassembly. Sci. Rep..

[23] Wenzel T. Variables That Influence Fluorescence Intensity. https://chem.libretexts.org/Bookshelves/Analytical_Chemistry/Molecular_and_Atomic_Spectroscopy_(Wenzel)/3%3A_Molecular_Luminescence/3.6%3A_Variables_that_Influence_Fluorescence_Measurements#:~:text=Therefore%2C%20fluorescent%20intensity%20is%20dependent,thereby%20reducing%20the%20fluorescent%20intensity.

[24] Lakowicz J.R., Lakowicz J.R. (2006). Solvent and Environmental Effects. Principles of Fluorescence Spectroscopy.

[25] Methylene Blue. https://ursabioscience.com/online-store/photosensitizers-for-1o2-production/methylene-blue-detail#:~:text=Methylene%20Blue%20is%20readily%20water,a%20quantum%20yield%20of%200.52.

[26] Yang R., Tang Z., Yan J., Kang H., Kim Y., Zhu Z., Tan W. (2008). Noncovalent Assembly of Carbon Nanotubes and Single-Stranded DNA: An Effective Sensing Platform for Probing Biomolecular Interactions. Anal. Chem..

[27] Li H., Zhang Y., Luo Y., Sun X. (2011). Nano-C60: A Novel, Effective, Fluorescent Sensing Platform for Biomolecular Detection. Small.

[28] Yu J., Yang L., Liang X., Dong T., Liu H. (2015). Bare Magnetic Nanoparticles as Fluorescence Quenchers for Detection of Thrombin. Analyst.

[29] Dabhi S.D., Roondhe B., Jha P.K. (2018). Nucleobases-Decorated Boron Nitride Nanoribbons for Electrochemical Biosensing: A Dispersion-Corrected DFT Study. Phys. Chem. Chem. Phys..

[30] Gu M.B., Kim H.-S. (2014). Biosensors Based on Aptamers and Enzymes.

[31] Trachman R.J., Truong L., Ferré-D’Amaré A.R. (2017). Structural Principles of Fluorescent RNA Aptamers. Trends Pharmacol. Sci..

[32] Wei Z., Wang J.J., Fultz L.M., White P., Jeong C. (2021). Application of Biochar in Estrogen Hormone-Contaminated and Manure-Affected Soils: Impact on Soil Respiration, Microbial Community and Enzyme Activity. Chemosphere.

[33] Tashyrev O., Prekrasna I. (2014). Express Method for Redox Potential and PH Measuring in Microbial Cultures. Int. J. Bioautomotion.

[34] Ragavan K.V., Selvakumar L.S., Thakur M.S. (2013). Functionalized Aptamers as Nano-Bioprobes for Ultrasensitive Detection of Bisphenol-A. Chem. Commun..

[35] Eisenhofer R., Minich J.J., Marotz C., Cooper A., Knight R., Weyrich L.S. (2019). Contamination in Low Microbial Biomass Microbiome Studies: Issues and Recommendations. Trends Microbiol..

