# Supplementary material for: Detecting Redox Potentials Using Porous Boron Nitride/ATP-DNA Aptamer/Methylene Blue Biosensor to Monitor Microbial Activities
Source: Micromachines (Basel). 2022 Jan 4;13(1):83. doi: 10.3390/mi13010083 (PMC8777636; doi:10.3390/mi13010083)
Supplement: Supplementary file 1 [file micromachines-13-00083-s001.zip › micromachines-1528308-supplementary.pdf]

Article

Detecting redox potentials using porous boron nitride/ATP-DNA aptamer/methylene blue biosensor to monitor microbial activities

Kai Guo, Zirui Song , Gaoxing Wang and Chengchun Tang

	1	2	3	4	5	6	7	8	9	10	11	12
A	100 a	100 a	90 a + 5b + 5c	90 a + 5b + 5c	90 a + 6b + 4c	90 a + 6b + 4c	90 a + 7b + 3c	90 a + 7b + 3c	90 a + 8b + 2c	90 a + 8b + 2c	90 a + 9b + 1c	90 a + 9b + 1c
B	80 a + 19b + 1c	80 a + 19b + 1	80 a + 18b + 2c	80 a + 18b + 2c	80 a + 17b + 3c	80 a + 17b + 3c	80 a + 16b + 4c	80 a + 16b + 4c	80 a + 15b + 5c	80 a + 15b + 5	80 a + 14b + 6c	80 a + 14b + 6c
C	80 a + 13b + 7c	80 a + 13b + 7c	80 a + 12b + 8c	80 a + 12b + 8	80 a + 11b + 9c	80 a + 11b + 9c	80 a + 10b + 10c	80 a + 10b + 10c	80 a + 9b + 11c	80 a + 9b + 11c	80 a + 8b + 12c	80 a + 8b + 12c
D	80 a + 7b + 13c	80 a + 7b + 13c	80 a + 6b + 14c	80 a + 6b + 14c	80 a + 5b + 15c	80 a + 5b + 15c	80 a + 4b + 16c	80 a + 4b + 16c	80 a + 3b + 17c	80 a + 3b + 17c	80 a + 2b + 18c	80 a + 2b + 18c
E	80 a + 1b + 19c	80 a + 1b + 19c	70 a + 29b + 1c	70 a + 29b + 1c	70 a + 28b + 2c	70 a + 28b + 2c	70 a + 27b + 3c	70 a + 27b + 3c	70 a + 26b + 4c	70 a + 26b + 4c	70 a + 25b + 5c	70 a + 25b + 5c
F	70 a + 24b + 6c	70 a + 24b + 6c	70 a + 23b + 7c	70 a + 23b + 7c	70 a + 22b + 8c	70 a + 22b + 8c	70 a + 21b + 9c	70 a + 21b + 9c	70 a + 20b + 10c	70 a + 20b + 10c	70 a + 19b + 11c	70 a + 19b + 11c
G	70 a + 18b + 12c	70 a + 18b + 12c	70 a + 17b + 13c	70 a + 17b + 13c	70 a + 16b + 14c	70 a + 16b + 14c	70 a + 15b + 15c	70 a + 15b + 15c	70 a + 14b + 16c	70 a + 14b + 16c	70 a + 13b + 17c	70 a + 13b + 17c
H	70 a + 12b + 18c	70 a + 12b + 18c	70 a + 11b + 19c	70 a + 11b + 19c	70 a + 10b + 20c	70 a + 10b + 20c	70 a + 9b + 21c	70 a + 9b + 21c	70 a + 8b + 22c	70 a + 8b + 22c	70 a + 7b + 23c	70 a + 7b + 23c

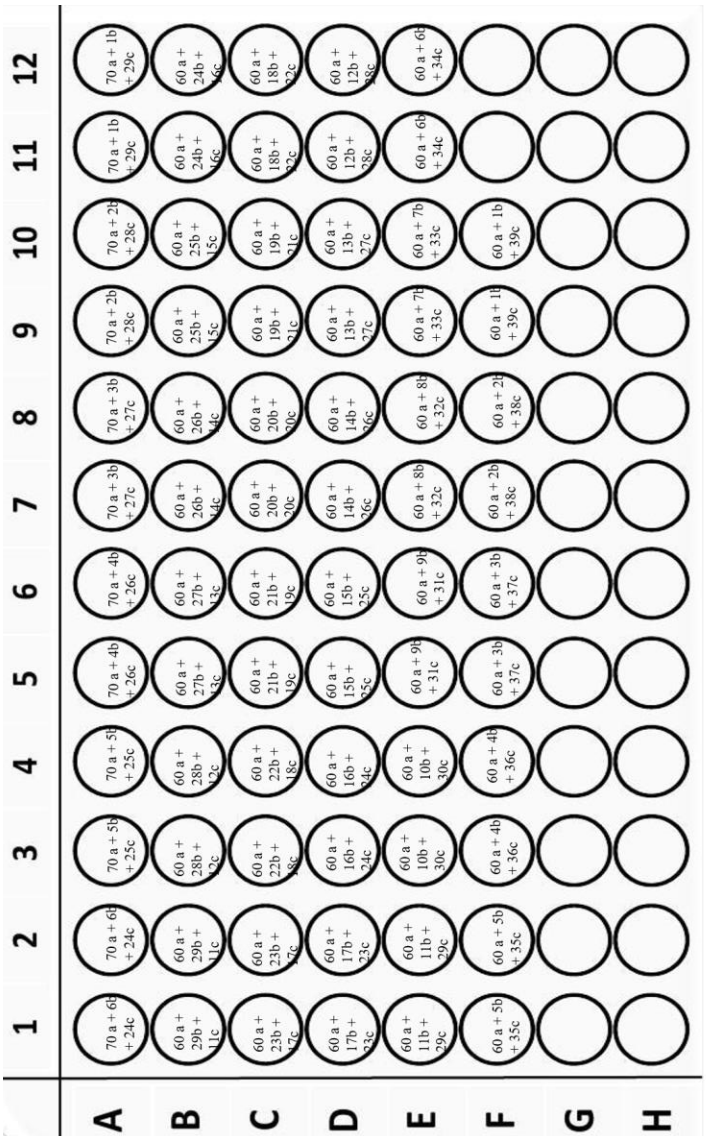

Figure S1. Fluorescence testing when changing the composition of microbial community (a: *Flavobacteria sp.*; b: *E coli.*; c: *Bacillus subtilis*).

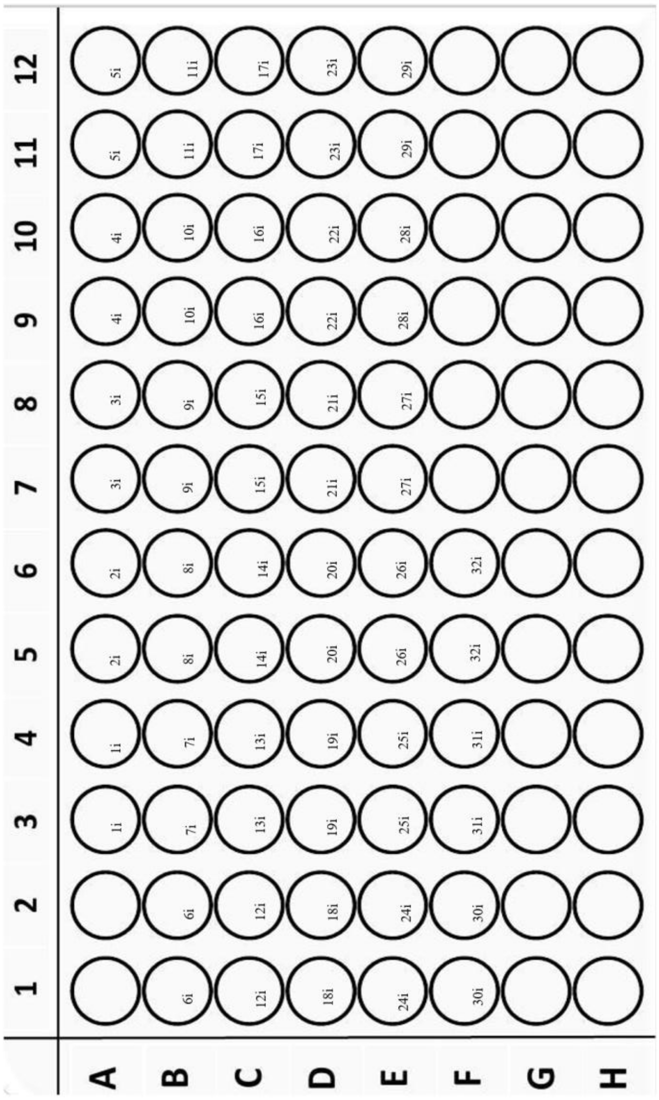

Figure S2. Added heavy metals to each well (Control A1 & A2). $Zn(CH_3COO)_2$ (i) is shown as an example here, according to which the other three metals are added as well.

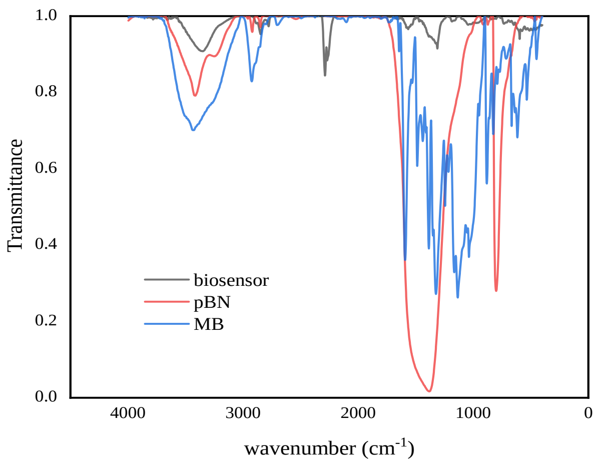

Figure S3. FTIR spectra of the biosensor, pBN and MB.

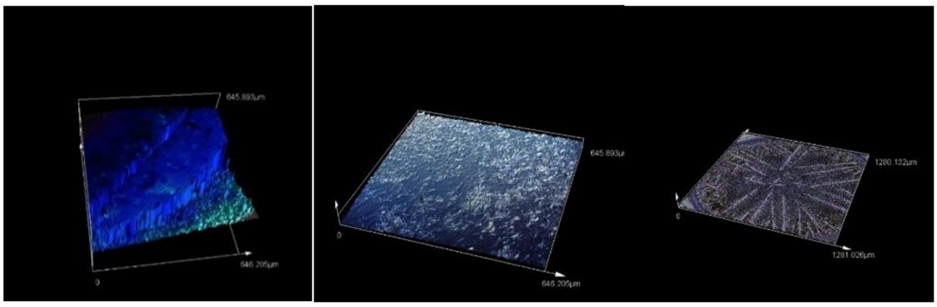

Figure S4. Images for biosensor (a), Flavobacterium sp. & biosensor (b), and Flavobacterium sp. & E coli. & Bacillus subtills (c) using laser bifocal microscope.

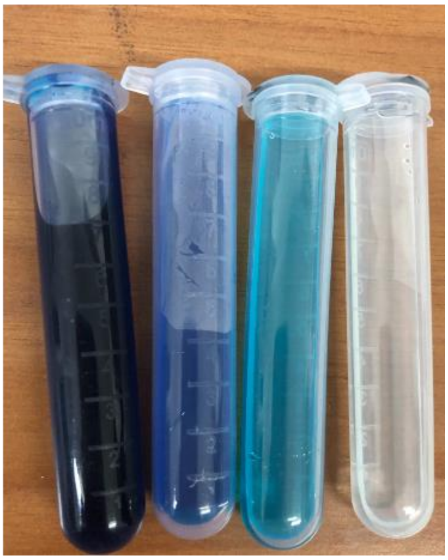

Figure S5. Colorimetric differences (a)MB; (b) MB-aptamer; (c) MB-aptamer-Fe (III); (d) MB-aptamer-ascorbic acid.

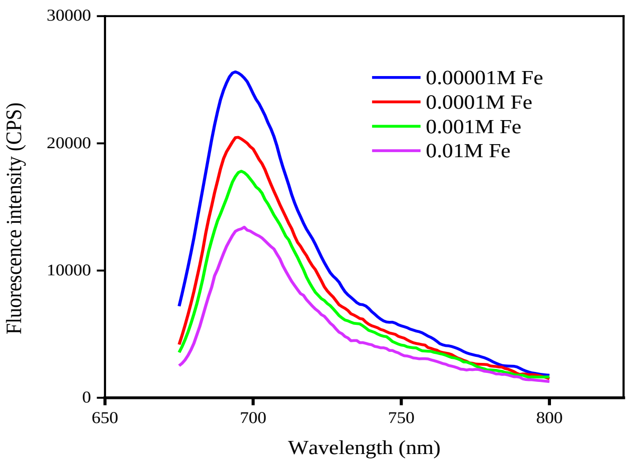

Figure S6. Fluorescence change when MB/biosensor was added to Fe (III) solution with different concentrations.

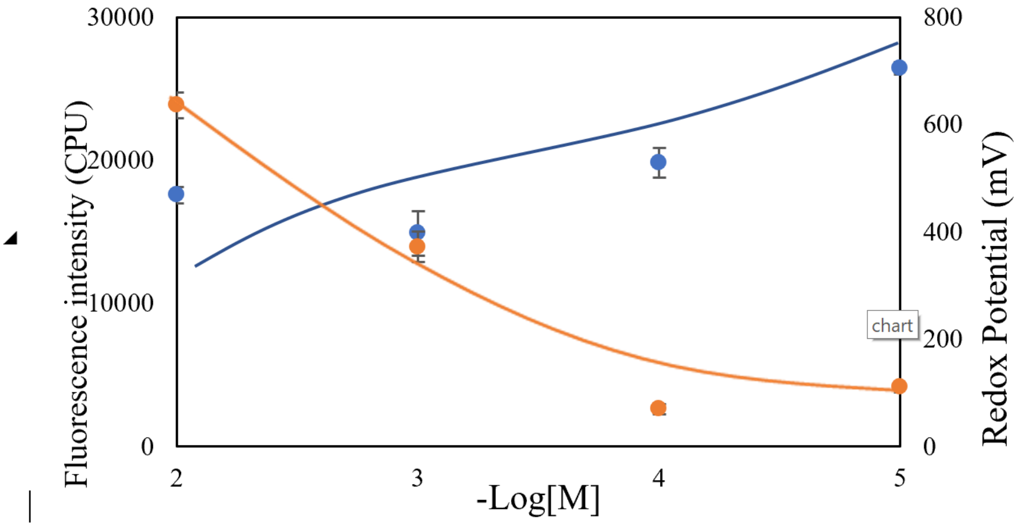

Figure S7. "Mirror" relationship between fluorescence intensity and redox potential as the concentration of Fe (III) inclined. .

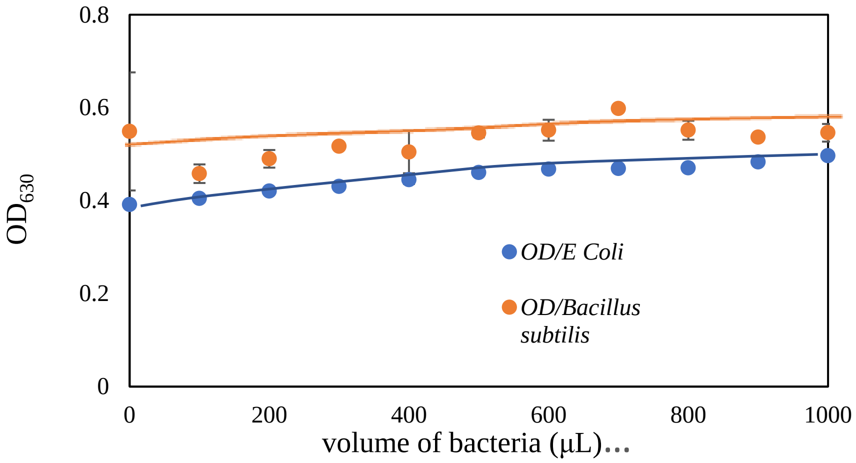

Figure S8. OD_{630} values after Flavobacteria sp. were grown with E coli. or Bacillus subtilis.

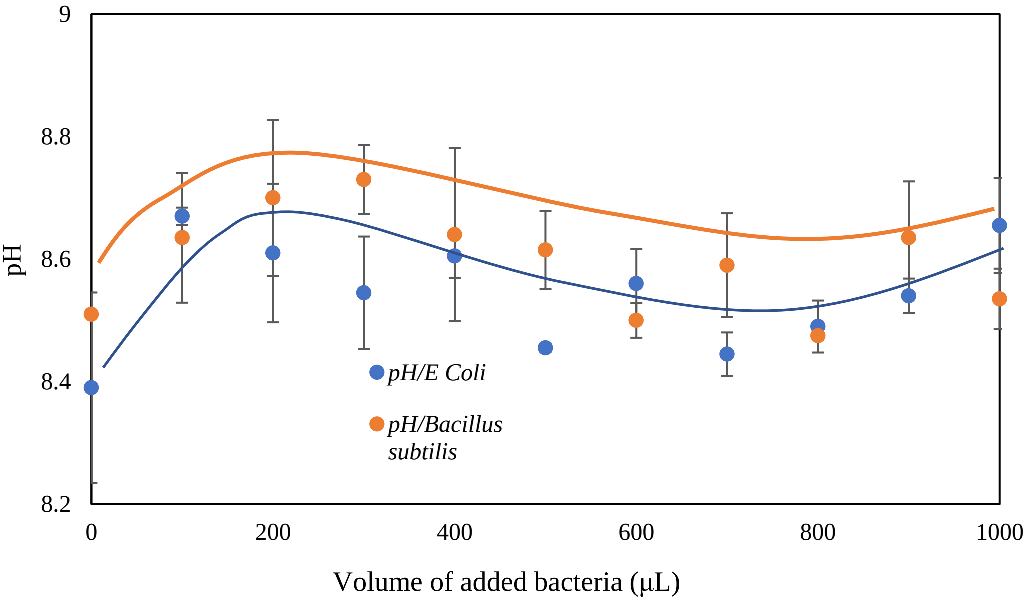

Figure S9. pH change after Flavobacteria sp. were grown with E coli. and Bacillus subtilis.
